# Quantifying Collective Attention from Tweet Stream

**DOI:** 10.1371/journal.pone.0061823

**Published:** 2013-04-30

**Authors:** Kazutoshi Sasahara, Yoshito Hirata, Masashi Toyoda, Masaru Kitsuregawa, Kazuyuki Aihara

**Affiliations:** 1 Graduate School of Information Science, Nagoya University, Nagoya, Japan; 2 Institute of Industrial Science, The University of Tokyo, Meguro-ku, Tokyo, Japan; 3 FIRST, Aihara Innovative Mathematical Modelling Project, Japan Science and Technology Agency, Meguro-ku, Tokyo, Japan; Bristol University, United Kingdom

## Abstract

Online social media are increasingly facilitating our social interactions, thereby making available a massive “digital fossil” of human behavior. Discovering and quantifying distinct patterns using these data is important for studying social behavior, although the rapid time-variant nature and large volumes of these data make this task difficult and challenging. In this study, we focused on the emergence of “collective attention” on Twitter, a popular social networking service. We propose a simple method for detecting and measuring the collective attention evoked by various types of events. This method exploits the fact that tweeting activity exhibits a burst-like increase and an irregular oscillation when a particular real-world event occurs; otherwise, it follows regular circadian rhythms. The difference between regular and irregular states in the tweet stream was measured using the Jensen-Shannon divergence, which corresponds to the intensity of collective attention. We then associated irregular incidents with their corresponding events that attracted the attention and elicited responses from large numbers of people, based on the popularity and the enhancement of key terms in posted messages or “tweets.” Next, we demonstrate the effectiveness of this method using a large dataset that contained approximately 490 million Japanese tweets by over 400,000 users, in which we identified 60 cases of collective attentions, including one related to the Tohoku-oki earthquake. “Retweet” networks were also investigated to understand collective attention in terms of social interactions. This simple method provides a retrospective summary of collective attention, thereby contributing to the fundamental understanding of social behavior in the digital era.

## Introduction

Behavior does not fossilize. This obvious fact has prevented us from exploring the dynamics and evolution of social behavior quantitatively, but the rise of online social media provides a new research possibility. In recent years, online social media have become a rapidly emerging communication vehicle, which readily allow people to transmit and share information in real time using PCs and mobile devices. For example, Twitter, one of the most popular social networking services, allows its users to post and read short text messages that discuss current events, known as “tweets,” which can contain no more than 140 characters. Thus, Twitter users behave as “social sensors” who actively sense real-life events and spontaneously voice their opinions, which are delivered immediately over user networks [1]. Because Twitter aggregates these messages over a long period of time and the data are publicly accessible via the Application Programming Interface (API), it makes available a massive “digital fossil” of real-time social interactions, which we can investigate to explore the temporal, spatial, and topical natures of social behavior in a quantitative manner at a fine degree of resolution. Therefore, online social data are valuable for testing existing hypotheses as well as for developing novel hypotheses or theories about social behavior.

Thus far, several aspects of Twitter have been reported in the literature, such as the structural properties of its user networks [2], [3], characteristics of online social interactions [4]–[6], dynamics of information diffusion [7], [8], and social dynamics with respect to particular real-life events [9], [10], as well as its availability for future trend prediction [11], [12] and sociological and cyber-psychological observations [13]–[17]. Moreover, several methodological studies have been conducted using Twitter data from theoretical and practical perspectives, including event detection [18], [19], the prediction of emerging topics [20], [21], and rumor identification [22].

In this study, we investigated the emergence of “collective attention” on Twitter. The attention of people is usually dispersed over a wide variety of concerns, but it can concentrate on particular events suddenly or gradually, and shift elsewhere very rapidly, which we refer to as collective attention. Our operational definition of collective attention is provided in the next section. Quantifying particular tweets that attract many users is the first step when exploring collective attention, although the rapid time-variant nature and large volumes of data make such quantification difficult. Furthermore, tweet conversations are usually overwhelmed by a flood of trivial messages, which can make meaningful analysis very difficult. A previous study on collective attention on Twitter focused on “hashtags,” which are attached by users to categorize topics of tweets. For example, #olympic was used for tweeting about the Olympic Games and #jishin was used for general earthquake information in Japanese. Spikes were detected in a hashtag series using a burst detection algorithm, and specific tweets related to people's attention were categorized into four classes of collective attention based on the temporal features of hashtag evolution [23].

In this study, we are interested in detecting collective attention and quantifying its intensity to compare different types of attention on the same basis. Furthermore, we assume that several types of attention are not categorized explicitly using hashtags. We also presume that collective attention does not always lead to a burst-like increase in tweet activity because it may appear as sustained or gradual activity throughout a day. Therefore, we consider an alternative approach by focusing on the distance between the regularity and irregularity of tweet streams. Any irregular incidents detected are then identified semantically by examining the popularity and the enhancement of key terms in tweets. Using a large dataset of Japanese tweets, we demonstrated the effectiveness of our proposed method for summarizing collective attention and made some key observations.

## Materials and Methods

### Tweet collection

We collected publicly available tweets between April 2011 and January 2012 by snowball sampling using Twitter REST API ver.1 [24]. First, we selected 10 seed users who had very large numbers of followers (i.e., celebrities) and collected a maximum of their most recent 3,200 tweets, which was the upper limit allowed by Twitter REST API ver.1. Subsequently, we collected tweets from other users who retweeted (i.e., forwarded) or replied to the tweets by the seed users. By considering all the collected users as new seed users, the same procedure was repeated several times. We also collected new tweets from all users, which were posted after the first collection procedure. The application of snowball sampling for a long period of time facilitates the collection of consistent longitudinal data that contain massive numbers of interactions among identified users, and this is an advantage compared with random sampling. This approach finally yielded a dataset that contained 493,001,412 tweets from 438,464 users (mostly Japanese). Although our dataset did not contain “official retweets,” which are created by hitting the retweet button on the Twitter website and its clients, it did contain so-called “unofficial retweets,” which were tweets retweeted with the abbreviation “RT” or “via.” Each tweet contained a text message and metadata, including the timestamp of the tweet, the user name and profile, and the geolocation, if available. We used the user name, text messages, and timestamps, which we converted to Japan Standard Time (JST) before analysis. Our dataset was a small subset of all Japanese tweets (n.b., as of July 2012, there were approximately 30 million active users in Japan and Japanese is the second most popular language on Twitter [25]), but our analysis showed that this dataset was sufficient to quantify collective attention reliably.

### Detection and measurement of collective attention

**Figure 1:**
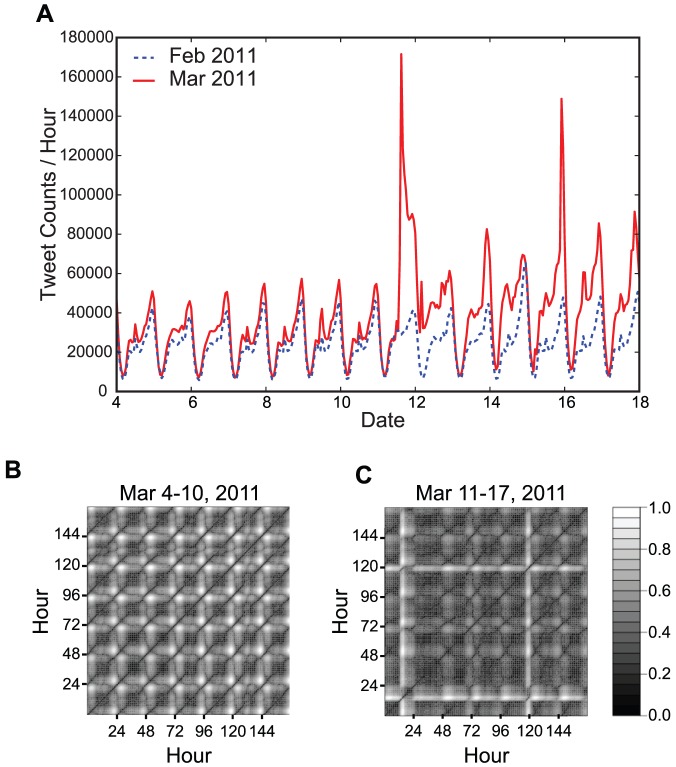
Regular and irregular states of the tweet stream. (A) Tweet count series for February and March 2011. Unthresholded recurrence plot [41] of the tweet time series (B) before the Tohoku-oki earthquake (March 4–10, 2011) and (C) after the earthquake (March 11–17, 2011). The gray scale corresponds to the distance between the tweet count series in a reconstructed phase space. Darker points indicate that two corresponding states are close to each other, whereas lighter points indicate that two corresponding states are distant from each other.

Our method was based on observations that the tweet activity obeys a regular circadian rhythm in normal situations with three relative peaks: one each in the early morning, noon, and late evening. By contrast, after a mega-event occurs in real life, the tweet stream tends to exhibit a burst-like increase and an unstable oscillation. Figure 1A shows an extreme example that contains abrupt spikes and a collapsed stream of tweets, which are thought to be associated with people's attention related to the Tohoku-oki earthquake and tsunami that struck Japan on March 11, 2011. We constructed the recurrence plots [26] for hourly tweet count series to visually examine the cyclostationarity of the tweet stream before and after the earthquake, where the embedding dimension we used was three and the time delay was one hour. Regular spatial patterns were observed from March 4–10 (Fig. 1B), whereas irregular spatial patterns occurred from March 11–17 (Fig. 1C). The recurrence plot shows the recurrence of states for the tweet count series in a reconstructed phase space (see Fig. 1 caption for details). These observations suggested that tweets related to particular events that attract many users could be distinguished from trivial ones by measuring the difference between the regular and irregular states of tweet stream. Therefore, we assumed that a large deviation from the circadian rhythm of the tweet stream would be associated with the emergence of collective attention on Twitter.

To measure this deviation, we compared the probability distributions of tweets on a daily basis using the Jensen-Shannon divergence (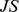), which is a symmetric version of the Kullback-Leibler divergence (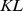) used to quantify (in bits) how similar a probability distribution 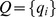 is to a model probability distribution 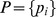, where 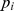 and 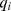 are the tweet probabilities at time index 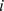. Unlike 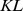, 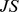 is always well defined and bounded. 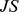 and 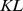 are defined as follows [27]:
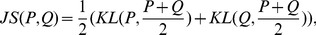
(1)

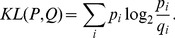
(2)


**Figure 2:**
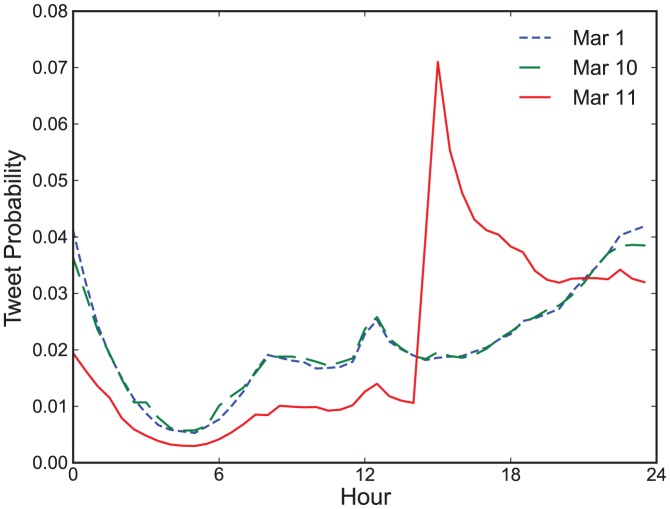
Daily tweet activity. Tweet probability distribution for March 11, 2011, the day of the Tohoku-oki earthquake, showing a burst-like increase and a sustained active state, whereas those for March 1 and 10 exhibit almost the same temporal profiles as those on normal days.

For example, Figure 2 compares three different probability distributions of tweets in 2011, where the number of tweets was counted every 30 min (i.e., 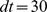): March 1, a weekday without any particular events; March 10, the day before the earthquake; and March 11, the day of the earthquake. As expected, March 10 had almost the same tweet probability distribution profile as March 1, which yielded a low 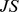 value (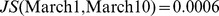). In contrast, the tweet probability distribution profile on March 11 was very different from that on March 1, yielding a high 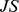 value (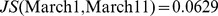). Thus, we can measure the level of deviation from a regular circadian rhythm in the tweet stream, which was assumed to be proportional to the intensity of the collective attention.

In the following measurements of 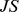 values, we used a daily probability distribution for each day of 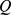 and its annual mean was 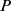. Note that the 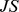 value had to be compared with data from the same year because the annual mean 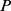 may change on an annual basis due to changes in lifecycle or other reasons (see Fig. S1 in Supporting Information (SI)).

### Semantic identification of collective attention

The popularity of terms (i.e., term frequency) in tweet texts provides useful information for identifying the contents of collective attention events. However, the ranking of the term frequency is normally dominated by a large amount of trivial terms that are not associated with particular events, e.g., “http” (used for URLs), “今日” (today), and “さん” (Mr. or Ms.) in Japanese tweets, whereas key terms that are less common but significant when characterizing corresponding events may be ignored. This situation is particularly likely to occur during moderate intensity collective attention events. Therefore, we introduced “popularity enhancement” to measure the increase in the term frequency rate after the detection of an irregular incident, which we define below, and we utilized this metric in addition to the popularity of terms.

We measured the term frequency in posted tweets (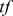) when the temporal profile of 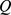 exceeded that of the annual mean 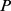 and we ranked all terms in descending order. The resulting ranking represented the popularity of terms. Furthermore, we computed 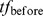 during the same time period on the previous day, or more than two days before in certain situations. Next, we collected the terms that met the condition 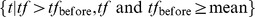, where 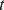 denotes a term. Using these terms, the popularity enhancement was computed as 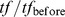. This condition has three important features. First, we use terms where 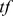 is greater than or equal to the mean frequency to avoid a situation where terms with a small 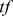 would abruptly increase on the next day because of a sampling problem or data sparseness, thereby causing a high popularity enhancement. Second, this condition filters out trivial terms because such terms always appear at an almost constant frequency, so 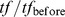 becomes almost one. Third, this condition also highlights key terms that increase drastically from the previous day, which are strongly associated with people's attention.

We used noun terms to compute the popularity and popularity enhancement to identify the contents of collective attention events. Our data were Japanese tweets, so before performing the aforementioned procedures, we conducted a morphological analysis of tweet texts to segment and extract noun terms from continuous sentences using MeCab, which is an open source Japanese morphological analyzer [28], based on NAIST-jdic, a Japanese dictionary [29]. We used this dictionary in its actual state and did not modify it to maximize the precision of the morphological analysis because this was outside the scope of this study. We demonstrate that our method worked for Japanese tweets; however, in principle, this semantic identification approach is language-independent and applicable to other languages. In the case of English tweets, for example, terms are already punctuated by spaces so one could skip the morphological analysis and follow the other procedures mentioned above.

## Results

**Figure 3:**
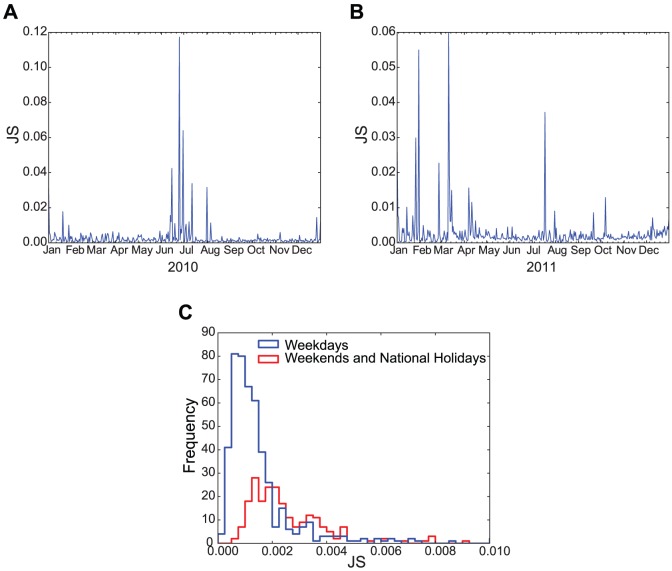
Distributions of the JS values related to collective attention. Intensity of collective attention (i.e., the 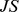 value) in (A) 2010 and (B) 2011. (C) Distribution of 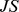 values on weekdays, weekends, and national holidays. Only 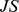 values 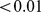 are shown.

We measured the 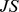 value on each day in the Japanese tweets we collected during 2010 and 2011. The results are shown in Figs. 3A and 3B where 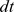 = 30 (min) for 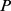 and 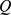 in Eqs. (1) and (2). For each day with a distinct 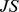 peak in these figures, we expected that there would be a large deviation from the regular tweet stream. In addition, Fig. 3C shows that the mean 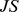 value for weekends and national holidays is significantly different from that of weekdays (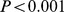; Mann-Whitney 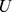 test), which indicated that the 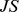 value was higher on non-working days with no events because of different life cycles. This characteristic was used to set the 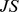 threshold (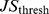) for collective attention candidates. The data from non-working days in Fig. 3C were fitted using a Gaussian function and the estimated mean and standard deviation of the function were 0.0019 and 0.0009, respectively. In the subsequent analysis, we focused on 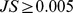, which was greater than 3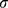 from the mean and statistically significant. In Figs. 3A and 3B, when 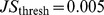, the numbers of irregular incidents detected were 34 in 2010 and 26 in 2011, and when 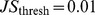, the numbers of irregular incidents detected were 17 in 2010 and 12 in 2011. Based on the popularity and the enhancement of key terms, we associated the detected incidents with the contents of the corresponding collective attention events by examining incidents in different categories, such as natural disasters, sporting events, culture, and annual regular events (see Tables S1 and S2 in SI for the complete lists).

**Figure 4:**
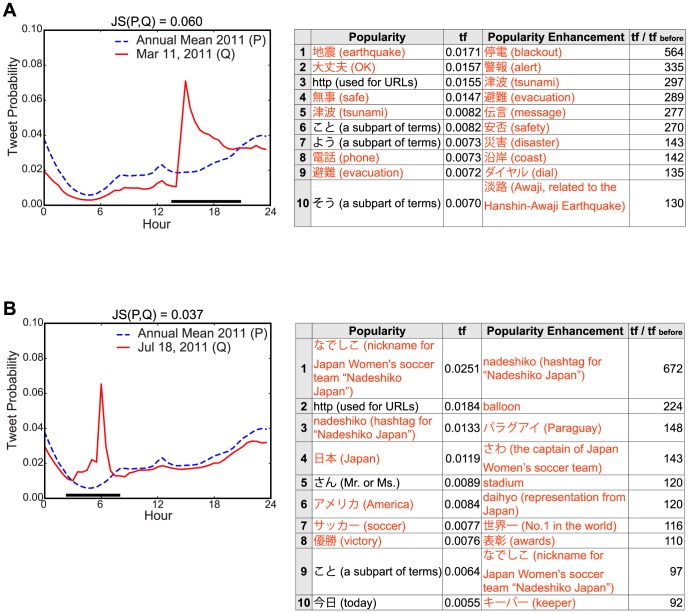
Examples of strong collective attention. Tweet probability distribution, and the rankings of the popularity and the enhancement of key terms in Japanese tweets on (A) March 11, 2011 and (B) July 18, 2011. The collective attention in (A) was associated with the Tohoku-oki earthquake and tsunami, whereas that in (B) was associated with the FIFA Women's World Cup, 2011. The black bars in the figures indicate the time period when the posted tweets were analyzed to compute term frequency. The red text in the tables denotes terms related to the target events and the text in parentheses shows the English translation.

As shown in Fig. 4, we found that strong collective attention events (i.e., a large 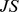 value) were associated with natural disasters and major sporting events. For example, the greatest attention in 2011, as shown in Fig. 2, emerged immediately after 14:46 on March 11, 2011, and the 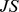 values were more than 0.005 for five consecutive days. This was the only case where the collective attention was maintained for such a long period of time. This long-term attention event appeared to be unusual given the highly volatile nature of people's attention, which normally decays within a few hours, as seen in other examples. Figure 4A shows that the attention focus on this day was undoubtedly the Tohoku-oki earthquake and the subsequent accidents because “地震” (earthquake) was the most frequent term while “停電” (blackout), “警告” (alarm), and “津波” (tsunami), which appear rarely in everyday tweet conversation, were used hundreds of times compared with the same time on the previous day. These quantitative results showed how strongly and continuously the Japanese people were affected by the Tohoku-oki earthquake and the subsequent social disorder. We may ask why “地震” (earthquake) was not the number one noun in terms of popularity enhancement in Fig. 4A (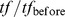 = 44; rank 34). This was because there were some large foreshocks before the Tohoku-oki earthquake so this noun was already in frequent use in tweet conversations. Other natural disasters such as aftershocks and typhoons also resulted in strong collective attention events (Tables S1 and S2 in SI). However, another type of strong collective attention was detected during major sporting events, including the FIFA World Cup, Japan professional baseball championship, and Vancouver Olympics (Tables S1 and S2 in SI). For example, on July 18, 2011, the Japan women's soccer team won the championship after a close game. As the game progressed, people posted statements describing their joy and despair and they cheered on the team. Popular terms were “
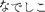
” (the nickname of the Japan women's soccer team) and “さわ” (the captain of the Japan women's team and the most valuable player in the tournament) (Fig. 4B). This was the third strongest collective attention event in 2011. A key observation was that, as expected, the popularity enhancement of key terms was successful for filtering trivial terms. However, strong attention was identified based only on the popularity of key terms.

**Figure 5:**
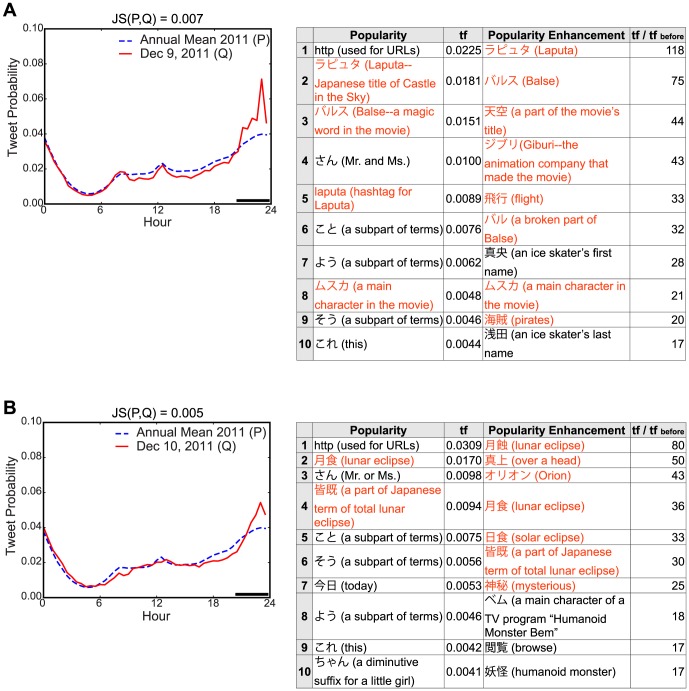
Examples of moderate collective attention. Tweet probability distribution, and the rankings of the popularity and the enhancement of key terms in Japanese tweets on (A) December 9, 2011 and (B) December 10, 2011. The collective attention in (A) was associated with the animated movie Castle in the Sky, whereas that in (B) was associated with a total lunar eclipse. The black bars in the figures indicate the time period when the posted tweets were analyzed to compute the term frequency. The red text in the tables indicates terms related to the target events and the text in parentheses shows the English translation.

A moderate 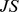 value was typically associated with some interesting types of collective attention, including culture, science, technology, and politics. These types include the return of Hayabusa (a spacecraft that collected samples from the surface of a near-Earth asteroid) to the Earth, the total lunar eclipse, televised animation movies, and early election reports (Tables S1 and S2 in SI). In particular, the observed attention event related to the animation movie “Castle in the Sky” was interesting from a social behavior perspective because when the central characters in the movie uttered the magic word “Balse!,” users tweeted “Balse!” simultaneously (Fig. 5A). There were no apparent instructions to make this tweet and no appreciable benefits, but users spontaneously exhibited synchronized tweets and the record for the most tweets per second (TPS) was set at that time [30]. In general, people were also interested in natural phenomena and science. Figure 5B shows that many people tweeted the terms “月蝕” or “月食” (both terms mean a lunar eclipse), even at midnight, to share atmospheric information related to the astronomical show, although some inadvertently tweeted “” (solar eclipse). There were also cases where multiple attention types emerged on the same day (Fig. S2 in SI). These examples confirmed that moderate collective attention was often related to interesting social behavior, including emerging novel cultures and customs on the Internet. Annual events such as New Year's Eve and New Year holidays were also subjects of moderate attention that had distinct tweet streams. For example, people habitually post greetings to friends and families at midnight on New Year's Eve in Japan, which led to a burst-like increase in tweets (Fig. S3 in SI). On the following one or two days people enjoy a holiday lifecycle, which led to a non-burst-like but unusual tweet increase throughout the day (Tables S1 and S2 in SI). Some high 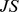 values were related to Twitter outages when users could not tweet because the Twitter service was down and the resulting tweet stream behaved differently (Fig. S2 in SI). Popularity enhancement usually provided more hints than the popularity itself in the semantic grounding of moderate attention, as shown in Fig. 5.

**Figure 6:**
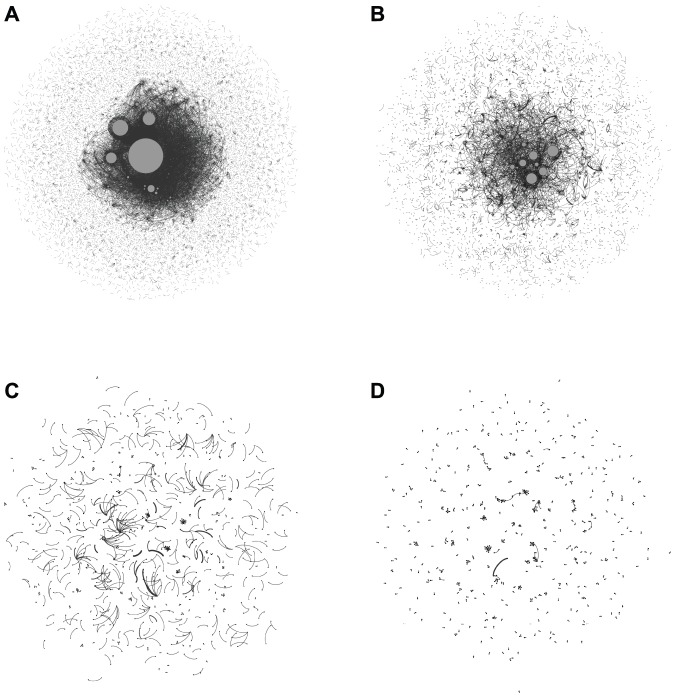
Visualization of RT networks during collective attention. (A) Tohoku-oki earthquake (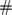 of nodes = 27,340, 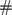 of links = 27,709), (B) FIFA Women's World Cup 2011 (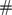 of nodes = 9,277, 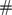 of links = 8,450), (C) Castle in the Sky (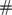 of nodes = 1,183, 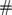 of links = 793), and (D) total lunar eclipse (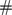 of nodes = 893, 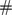 of links = 553). The nodes represent users, which are connected if there is a RT with key terms related to the target event. In each figure, the node sizes are proportional to the number of retweeted tweets. Only the nodes with 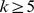 retweeted tweets are shown for clarity.

**Figure 7:**
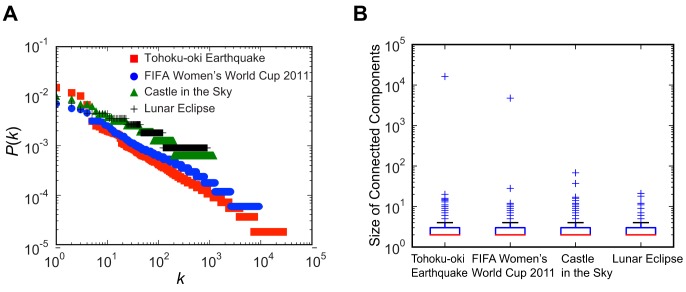
Structural features of RT networks. (A) Degree distribution of the RT networks related to the Tohoku-oki earthquake, FIFA Women's World Cup, 2011, Castle in the Sky, and total lunar eclipse. (B) Boxplots of the connected component sizes in the RT networks.

We also considered collective attention in terms of social interactions by constructing retweet (RT) networks where the nodes represented users and pairs of nodes were linked if a retweet included key terms relevant to the target event. If user-A retweeted user-B's tweet in the form of “RT @user-B…” or “via @user-B…” they were linked as follows: A
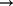
B (a tweet origin). Multiple tweet origins were possible for user-A's retweet such as “RT @user-B… RT @user-C…” which resulted in links as follows: A
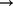
B and A
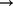
C. The RT networks were constructed and analyzed using the same data and the key terms (shown in red) as those used in Figs. 4 and 5. Figure 6 shows examples of the RT networks for: (A) the Tohoku-oki earthquake, (B) the FIFA Women's World Cup, 2011, (C) Castle in the Sky, and (D) the lunar eclipse. The structures of these networks depended on the type of attention. Some nodes had dense connections with strong attention, such as (A) and (B), whereas most of the nodes had sparse connections with moderate attention, such as (C) and (D). These characteristics are quantified in Fig. 7. Figure 7A shows that all the RT networks exhibited a power law feature, although the strong and moderate collective attention events exhibited distribution ranges with different degrees. As shown in Fig. 7B, the size distribution of connected components also differed significantly. The RT networks of strong attention, i.e., (A) and (B), had a very large connected component, which we refer to as the “RT core,” whereas those with moderate attention, i.e., (C) and (D), lacked the RT core. We also computed the tweet enhancement level as the ratio of the number of tweets, using the key terms from the event day relative to those from the previous day, and the RT enhancement as the ratio of the number of RTs using the key terms from the event day relative to those from the previous day, using the same data and the key terms mentioned above. We found that the tweet and RT enhancement increased significantly, as follows: (A) 121 and 117, (B) 64 and 29, (C) 103 and 5, and (D) 24 and 18. These enhancements would have been unlikely to occur in normal conditions. This demonstrates that the key terms captured significantly greater attention on the days of the events. These findings suggest that a distinct social behavior underlies collective attention. Thus, a large burst of solo tweets and the diffusion of RTs that engaged many users determined the nature and intensity of collective attention.

## Discussion

We demonstrated that our proposed method was effective for quantifying the active reactions of users to real-life events, most of which were closely related to collective attention on Twitter. It is important to clarify the difference between the epidemic spread of information online and collective attention. Both can originate from external events to induce distinct tweet streams, although in different ways. The epidemic spread of information on Twitter involves a cascade of tweets and retweets over user networks. By contrast, collective attention differs depending on its degree where strong collective attention is accompanied by chains of retweets, as shown in Figs. 6A and 6B, as well as a large number of solo tweets, whereas moderate attention is induced mainly by a massive amount of solo tweets rather than retweets, as shown in Figs. 6C and 6D. The former process is equivalent to the epidemic spread of information, whereas the latter process is unique to collective attention. There is also a crucial difference in their topical nature, i.e., collective attention can be related to the mood or sentiment of users at the collective level, rather than simply information, as shown in our results. Therefore, the incidents detected using our method are better understood in the context of collective attention.

**Figure 8:**
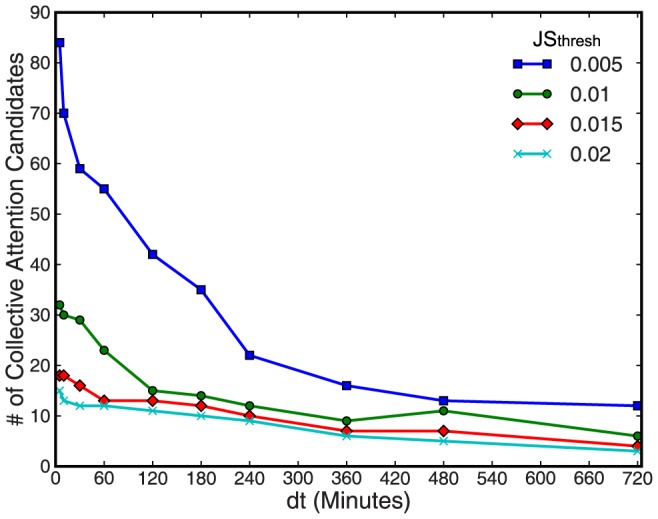
Parameter dependence during the detection of collective attention candidates. Smaller values of 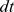 and 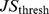 were correlated with the detection of more collective attention candidates. However, many false positive candidates were detected when these parameters were very small.

We also compared our method and observations with other related studies. First, several methods that utilize tweet streams have been proposed for event detection. A standard approach is to focus on a burst-like increase in tweets to detect significant events [23]. By contrast, our novel approach measured the overall difference between regular and irregular tweet streams. Therefore, our method could detect burst-like increases as well as non-burst-like increases with unusual tweet patterns, although we only encountered a few incidents of this type during our observation period (e.g., the second day of the New Year holidays). Several approaches have also used word-based features as the inputs for statistical methods or machine learning techniques [18], [19]. For longitudinal data, however, the extraction of words from tweets is a computationally intensive procedure in languages such as Japanese. Instead, we computed the tweet counts to determine the 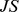 values of all the tweets to detect event candidates, before applying natural language processing to the selected tweets. This simple approach is expected to work well with longitudinal data in a feasible amount of time and it is applicable to other languages. However, this method has a possible disadvantage. If multiple significant events occur at the same time, it will be difficult to distinguish them in the rankings of the popularity and the enhancement of key terms. We could overcome this problem if we consider the locality of data during the analysis, for example, by limiting the geolocation of tweets or by focusing on particular groups where users share their interests. During the practical application of this method, it is important to consider the following properties. As shown in Fig. 8, the number of collective attention candidates increases as 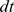 and 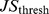 decrease. Thus, false positive irregular incidents will be encountered increasingly if these parameters are very small. Therefore, 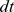 and 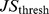 must be set appropriately by reference to the distribution of 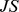 values of non-working days, as mentioned above.

Second, previous studies have used different approaches to focus on the tweet stream as a “mirror of reality,” such as using Twitter as a social sensor [1], [19], for estimating public mood [13], [15], [17], and for determining user attention based on hashtags [23]. In addition to the tweet stream, we investigated RT networks to elucidate the roles of social interactions during collective attention. We found that a type of collective attention could be understood from the perspective of social influence. According to the influentials theory [31], it is likely that a minority of users, known as influentials, are far better at spreading information than the majority of ordinary users. However, some studies have shown that this is not always the case and information cascades depend on how ordinary users interact and their situations, according to simulations [32] and empirical data analyses [33], [34]. The RT networks provided another example to support this notion, because they suggested that every ordinary user had the potential to become an influential. We observed that ordinary users could become influentials by posting tweets that captured attention. For example, during the Tohoku-oki earthquake emergency, the most retweeted user in our dataset (i.e., the largest node in Fig. 6A) was an ordinary user with approximately 200 followers, who temporarily became an influential by tweeting about the lessons of the Hanshin-Awaji Earthquake that occurred in Japan in 1995 (“淡路” (Awaji) was ranked in the list shown in Fig. 4A). This phenomenon can also be considered as a “wisdom of crowds” effect [35]. Thus, despite an overwhelming number of tweets, most of which were noise, beneficial tweets went viral via spontaneous RT chains by a variety of ordinary users, which we refer to as “social filtering.” Thanks to the high heterogeneity of users as information sources and social filtering, the wisdom of crowds effect can work on Twitter, although it depends on the degree of social influence, e.g., the accessibility of public information [36] or the type of public information available [37].

In conclusion, we developed a simple method for quantifying collective attention from tweet stream, which allowed us to compare different types of attention using the same metrics. We used this method to analyze Japanese tweets over a period of two years and identified strong collective attention during natural disasters and major sporting events, as well as moderate attention related to culture, science, technology, politics, and annual regular events. The category and intensity of people's attention may differ among languages, cultures, and societies, which is also true of their underlying social interactions (e.g., RT networks). Online social data in a digitized form can provide new insights into the dynamics and evolution of social behavior [38]–[40] so quantifying this data, as achieved in this study, is becoming increasingly important. Our simple approach does not have a predictive capacity but it takes full advantage of the tweet stream to produce a retrospective summary of collective attention, which can be used to further understand social behavior in the digital era.

## Supporting Information

Figure S1 **Annual variation in daily tweet activity.**
(PDF)

Figure S2 **Collective attention related to multiple events.**
(PDF)

Figure S3 **Collective attention related to new year holidays.**
(PDF)

Table S1 **Collective attention in 2010.**
(PDF)

Table S2 **Collective attention in 2011.**
(PDF)
