# Correction: Quantifying Collective Attention from Tweet Stream

**DOI:** 10.1371/annotation/25b6b59d-c3d4-4f9a-b14c-d8d6c389bda8

**Published:** 2013-05-17

**Authors:** Kazutoshi Sasahara, Yoshito Hirata, Masashi Toyoda, Masaru Kitsuregawa, Kazuyuki Aihara

There was an error in the funding statement. The correct funding information is:

This research is supported by the Japan Society for the Promotion of Science through the “Funding Program for World-Leading Innovative R&D on Science and Technology (FIRST Program),” initiated by the Council for Science and Technology Policy (CSTP). The funders had no role in study design, data collection and analysis, decision to publish, or preparation of the manuscript.

The legends for the Supporting Information figures were omitted. The full titles and legends for the Supporting Information Figures are as follows:

**Fig. S1. Annual variation in daily tweet activity.** Mean tweet probability distribution in 2010 (P) and 2011 (Q). JS(P, Q)=0.0009.

**Fig. S2. Collective attention related to multiple events.** Tweet probability distribution, and the rankings of the popularity and the enhancement of key terms in Japanese tweets on Jun 13, 2010. Each black bar in the figure denotes the time range during which tweets were considered for calculating term frequency. The red text in the tables denotes terms related to (A) an earthquake and (B) Hayabusa's return to the Earth. (C) in the figure is related to Twitter outage. The text in parentheses denotes the English translation.

**Fig. S3. Collective attention related to new year holidays.** Tweet probability distribution, and the rankings of the popularity and the enhancement of key terms in Japanese tweets on January 1, 2011. The black bar in the figure represents the time range during which tweets were considered for calculating term frequency. The red text in the table represent terms related to new year holidays, and the text in parentheses denotes the English translation. 

