# Supplementary figures and images for: Quantifying Collective Attention from Tweet Stream
Source: PLoS One. 2013 Apr 30;8(4):e61823. doi: 10.1371/journal.pone.0061823 (PMC3640043; doi:10.1371/journal.pone.0061823)

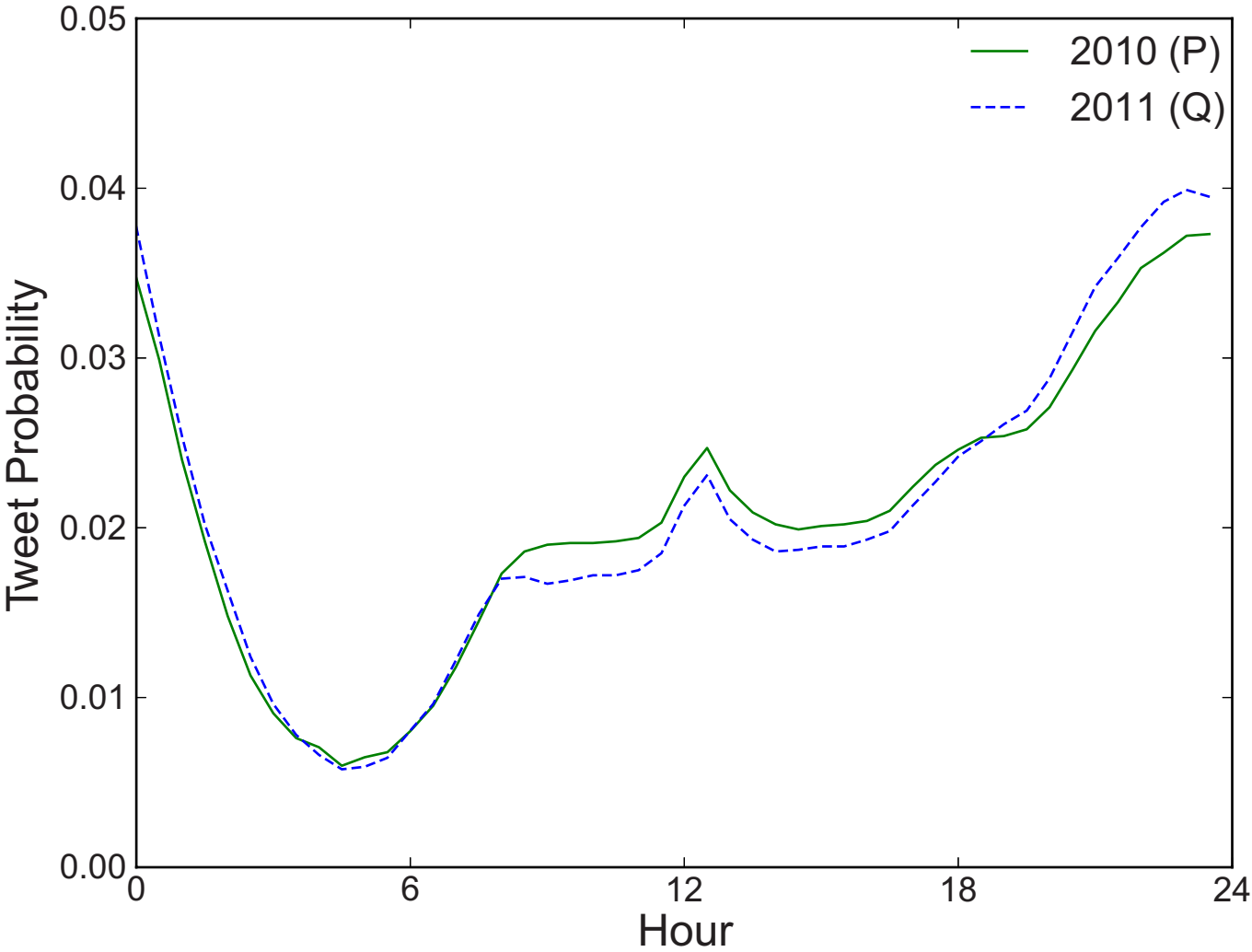

Supplement: Figure S1 — Annual variation in daily tweet activity. (PDF) [file pone.0061823.s001.pdf]
