# Supplementary material for: Quantifying Collective Attention from Tweet Stream
Source: PLoS One. 2013 Apr 30;8(4):e61823. doi: 10.1371/journal.pone.0061823 (PMC3640043; doi:10.1371/journal.pone.0061823)
Supplement: Figure S2 — Collective attention related to multiple events. (PDF) [file pone.0061823.s002.pdf]

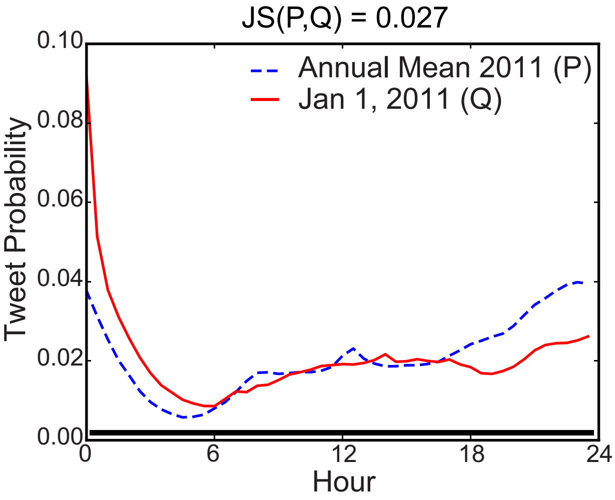

	Popularity	tf	Popularity Enhancement	tf / tf before
1	http (used for URLs)	0.0292	あけ (a subpart of New year's greetings)	259
2	今年 (this year)	0.0284	asamadetv (hashtag for a late-night TV program)	236
3	お願い (wishing)	0.0144	謹賀 (a subpart of New year's greetings)	74
4	さん (Mr. or Ms.)	0.0111	運勢 (luck)	56
5	こと (a subpart of terms)	0.0097	大吉 (best luck)	38
6	よう (a subpart of terms)	0.0072	学問 (studies)	36
7	新年 (new year)	0.0071	YEAR	36
8	twitpic (hashtag for Twitpic, a website for sharing picture and videos)	0.0051	CDTV (hashtag for a late-night TV program)	35
9	そう(a subpart of terms)	0.0042	願望 (wish)	35
10	今日 (today)	0.0037	くじ (lot)	32
