# Supplementary material for: Quantifying Collective Attention from Tweet Stream
Source: PLoS One. 2013 Apr 30;8(4):e61823. doi: 10.1371/journal.pone.0061823 (PMC3640043; doi:10.1371/journal.pone.0061823)
Supplement: Figure S3 — Collective attention related to new year holidays. (PDF) [file pone.0061823.s003.pdf]

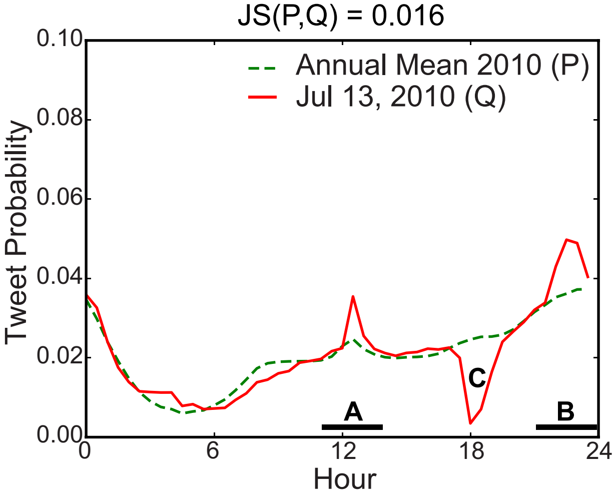


**A**

| | Popularity | tf | Popularity Enhancement | tf / tf before |
|---|---|---|---|---|
| 1 | http (used for URLs) | 0.0287 | 地震 (earthquake) | 122 |
| 2 | こと (a subpart of terms) | 0.0098 | 福島 (Fukushima, the earthquake center) | 17 |
| 3 | さん (Mr. or Ms.) | 0.0094 | 弟子 (pupil) | 16 |
| 4 | 地震 (earthquake) | 0.0093 | 目線 (eye line) | 11 |
| 5 | 今日 (today) | 0.0081 | Hayabusa (a Japanese space craft) | 8 |
| 6 | twitpic (hashtag for Twitpic, a website for sharing pictures and videos) | 0.0065 | 速報 (flash report) | 8 |
| 7 | そう (a subpart of terms) | 0.0051 | 日曜日 (Sunday) | 8 |
| 8 | よう (a subpart of terms) | 0.0050 | 気象庁 (the Meteorological Agency) | 7 |
| 9 | 自分 (myself) | 0.0035 | 四国 (Shikoku, one of the four main islands of Japan) | 7 |
| 10 | これ (this) | 0.0033 | kenta (a person’s name) | 6 |

**B**

| | Popularity | tf | Popularity Enhancement | tf / tf before |
|---|---|---|---|---|
| 1 | http (used for URLs) | 0.0340 | iOgd (a part of Ustream URL where Hyabusa’s return was live broadcasted) | 223 |
| 2 | live | 0.0156 | universe | 169 |
| 3 | こと (a subpart of terms) | 0.0108 | Hayabusa | 43 |
| 4 | さん (Mr. or Ms.) | 0.0088 | 安藤 (Ando, an ice skater’s name, Miki Ando) | 41 |
| 5 | ustre (a broken part of Ustream, a website for live video streaming of events online) | 0.0087 | ハヤブサ (Hayabusa) | 39 |
| 6 | universe | 0.0068 | hayabusa (hashtag for Hayabusa’s return) | 37 |
| 7 | iOgd (a part of Ustream URL where Hyabusa’s return was live broadcasted) | 0.0067 | 大陸 (a subpart of a TV program’s name) | 35 |
| 8 | 今日 (today) | 0.0066 | 情熱 (a subpart of a TV program’s name) | 30 |
| 9 | hayabusa (hashtag for Hayabusa’s return) | 0.0055 | ustream (a website for live video streaming of events online) | 26 |
| 10 | そう (a subpart of terms) | 0.0052 | 拍手 (clapping) | 24 |
