# Supplementary material for: Quantifying Collective Attention from Tweet Stream
Source: PLoS One. 2013 Apr 30;8(4):e61823. doi: 10.1371/journal.pone.0061823 (PMC3640043; doi:10.1371/journal.pone.0061823)
Supplement: Table S1 — Collective attention in 2010. (PDF) [file pone.0061823.s004.pdf]

Table S 1: **Collective attention in 2010.**

Date	JS	Content	Category
2010/1/1	0.032	New Year and holiday (New year greetings and resolutions)	Annual events
2010/1/2	0.006	New Year and holiday	Annual events
		Hakone Ekiden: First half	Sporting events
2010/1/9	0.010	All-Japan high school soccer tournament	Sporting events
2010/1/20	0.018	Twitter outage	Twitter outage
2010/1/28	0.010	Introduction of iPad	Technology
2010/2/13	0.006	Opening ceremony of the Vancouver Olympics	Sporting events
2010/2/26	0.006	Women's figure skating at the Olympics	Sporting events
2010/3/14	0.005	Earthquake (M6.6)	Natural disasters
2010/3/18	0.005	Twitter outage	Twitter outage
2010/3/21	0.005	Rainstorm	Natural disasters
2010/3/28	0.006	IT-related event	Technology
2010/4/5	0.006	Twitter outage	Twitter outage
2010/5/30	0.007	International friendship football: England beat Japan	Sporting events
2010/6/10	0.006	Release of a new hamburger	Culture
2010/6/13	0.016	Earthquake (M6.2)	Natural disasters
		Twitter outage	Twitter outage
		Hayabusa's return to the Earth	Science
2010/6/14	0.010	FIFA World Cup: Japan beat Cameroon	Sporting events
2010/6/15	0.042	Same as above	Sporting events
		Twitter outage	Twitter outage
2010/6/19	0.011	FIFA World Cup: Netherlands beat Japan	Sporting events
2010/6/25	0.117	FIFA World Cup: Japan beat Denmark	Sporting events
2010/6/27	0.008	FIFA World Cup: Germany beat England	Sporting events
2010/6/28	0.005	Same as above	Sporting events
2010/6/29	0.010	FIFA World Cup: Paraguay beat Japan	Sporting events
2010/6/30	0.064	Same as above	Sporting events
2010/7/3	0.008	FIFA World Cup: Quarter finals	Sporting events
2010/7/4	0.011	Same as above	Sporting events
2010/7/8	0.012	FIFA World Cup: Spain beat Germany in the semifinals	Sporting events
2010/7/11	0.005	Early report of the 22nd Upper House election	Politics
2010/7/12	0.030	FIFA World Cup: Spain beat Netherlands to win the cup	Sporting events
2010/8/1	0.032	Twitter outage	Twitter outage
2010/8/6	0.011	Twitter outage	Twitter outage
2010/10/8	0.005	International friendship football: Japan beat Argentina	Sporting events
		Animation movie "Lupin III: Castle of Cagliostro"	Culture
2010/11/7	0.006	Final game of the Japanese baseball championship	Sporting events
2010/12/26	0.015	M-1 Grand Prix, an annual comedy contest	Culture
2010/12/31	0.012	New Year's Eve	Annual events
