# Supplementary material for: Quantifying Collective Attention from Tweet Stream
Source: PLoS One. 2013 Apr 30;8(4):e61823. doi: 10.1371/journal.pone.0061823 (PMC3640043; doi:10.1371/journal.pone.0061823)
Supplement: Table S2 — Collective attention in 2011. (PDF) [file pone.0061823.s005.pdf]

Table S 2: **Collective attention in 2011.**

Date	JS	Content	Category
2011/1/1	0.027	New Year and holiday (New year greetings and resolutions)	Annual events
2011/1/2	0.008	New Year and holiday	Annual events
		Hakone Ekiden: First half	Sporting events
2011/1/3	0.007	New Year and holiday	Annual events
		Hakone Ekiden: Second half	Sporting events
2011/1/14	0.010	AFC Asian Cup: Japan beat Shiria	Sporting events
2011/1/22	0.008	AFC Asian Cup: Japan beat Qatar	Sporting events
		Successful launch of Kounotori 2 (a robot cargo spaceship)	Science
2011/1/25	0.006	AFC Asian Cup: Japan beat South Korea	Sporting events
2011/1/26	0.030	Same as above	Sporting events
2011/1/30	0.055	AFC Asian Cup: Japan beat Australia to win the cup	Sporting events
2011/2/26	0.023	Twitter outage	Twitter outage
2011/3/11	0.060	Tohoku-oki earthquake (M9.0) and tsunami	Natural disasters
2011/3/12	0.019	Afterquake (M6.6)	Natural disasters
2011/3/13	0.006	Afterquake (M6.0)	Natural disasters
2011/3/14	0.006	Afterquake (M6.2)	Natural disasters
2011/3/15	0.015	Afterquake (M6.0)	Natural disasters
2011/4/7	0.016	Earthquake (M7.4)	Natural disasters
2011/4/8	0.007	Same as above	Natural disasters
2011/4/11	0.012	Earthquake (M7.1)	Natural disasters
2011/4/12	0.007	Earthquake (M6.3)	Natural disasters
2011/4/16	0.006	Earthquake (M5.9)	Natural disasters
2011/7/18	0.037	FIFA Women's World Cup: Japan beat USA to win the cup	Sporting events
2011/7/31	0.009	Earthquake (M6.4)	Natural disasters
2011/9/21	0.009	Typhoon No.15	Natural disasters
2011/10/7	0.013	Twitter outage	Twitter outage
2011/12/9	0.007	Animation movie "Castle in the Sky"	Culture
2011/12/10	0.005	Total lunar eclipse	Science
2011/12/31	0.007	New Year's Eve	Annual events
